# Wonders of Plant Life

**Published:** 1888-04

**Authors:** 


### WONDERS OF PLANT LIFE.

A world of curiosities is revealed in the arboreal kingdom. The Banyan tree is said to be capable of sheltering not less than seven thousand men, and is still continuing to extend its branches over new territory. The roots spring from the limbs of the tree, and, falling like ropes, they enter the ground, and in process of time become thick, stout pillars, each tree forming a grove by itself.

The Baobab or Monkey-bread tree is another most extraordinary production of nature. Imagine to yourself a tree, thirty feet in diameter at the base, and only forty feet high, with the trunk rapidly diminishing toward the top, and then spreading out into what looks like a little forest. In one of the old trees the branches form a spherical head a hundred to a hundred and fifty feet in diameter, the center branch rising to the height of sixty feet, while others drop over the main trunk, and conceal it from view. Some of these trees have been hollowed out, and a space made large enough to hold twenty to thirty men, without any apparent injury to the tree. The Baobab must be the slowest growing plant in the world, as it is supposed to be one of the oldest. A tree has been cultivated in the gardens at Kew over forty years, and thus far it has attained the height of only four and a half feet. Some of these trees are estimated to be five thousand years old, and dates are cut in the bark which were made in the fourteenth century.

Early travelers in China and Tartary speak of a "plant of flesh and blood, with the shape and appearance of a lamb, having feet, head and tail distinctly formed, and its skin covered with soft down." The lamb is said to grow upon a stalk three feet high, and to turn about and bend to the herbage which serves for its food ; and when the grass falls, it dries up and withers away. There is some foundation for this queer story in the existence of a singularly shaped plant, with a sort of wooly covering ; and, in order to heighten the general effect, the natives trim the plant, and adjust the long, light, silky hairs that cover it in such a style as to give it the appearance of a wool-clad animal.

A slender, erect shrub grows in India, to which the name of the Telegraph plant has been given, because of a resemblance to railway telegraph signals in the motion of the trifoliate leaves, the two side ones rising and falling alternately for a time, when they rest for a season, and being most active in the early morning. Sometimes, many of the leaves may be seen in action at once, and then again only a few seem to be inspired with the notion, which shows that their action does not depend upon the wind.

The value of the plant world, not only in furnishing the pulpy material of which paper is manufactured, but also in supplying the paper itself outright, may not be fully appreciated by all our readers. It is well known that the Egyptian papyrus, in early days, was the main source from which anything like our modern paper was derived. This was a reed that grew by the brooks, with stem six to ten feet high, and about an inch in diameter. These were peeled, the pith cut into thin slices, which were then laid side by side, with the edges touching one another. After being sprinkled with water, a heavy pressure was applied, and they were thus united into one piece. It may not be so generally known that there now grows in Asia a tree the bark of which is made into sheets about a yard square, and these are used for all the ordinary purposes of paper, being very tough and durable. The soft and beautiful Chinese rice-paper is not the product of any part of the rice-plant, but it is the pith of a tree which, by the aid of a lathe and sharp instrument, is cut into very thin and delicate rolls.

These are a few specimens of the rarer curiosities of the plant world, but the list might be extended almost indefinitely.—*Banner of Life.*

